# Sustainable infrared-driven deposition of palladium nanoparticles on viscose for multi-functional textile engineering

**DOI:** 10.1186/s13065-025-01690-0

**Published:** 2025-12-10

**Authors:** Sahar A. El-Kholy, Maher H. Helal, Hossam E. Emam

**Affiliations:** 1 Chemistry Department, Faculty of Science, Helwan University, Ain-Helwan, Cairo, 11795 Egypt; 2 Department of Pretreatment and Finishing of Cellulosic Fibers, Textile Research and Technology Institute, National Research Centre, Scopus affiliation ID 60014618, 33 EL Buhouth St., Dokki, 12622 Giza Egypt

**Keywords:** PdNPs, In-situ, Infrared, Durable, Antimicrobial, UV protection

## Abstract

Prolonged exposure to ultraviolet (UV) irradiation and microbial contamination correlated to the environmental pollution and ozone layer depletion caused serious health concerns. Nanotechnology offers promising solutions in functional textiles, particularly through nanoparticles with high surface energy, ionization capacity, and surface area. Herein, for the first time the exploitation of infra-red irradiation for controllable nucleation of PdNPs was investigated. This study is considered with the enhancement of UV protection and antimicrobial properties of viscose fabrics via infrared (IR)-assisted/in-situ self-clustering of palladium nanoparticles (PdNPs). PdNPs were synthesized within viscose matrix under varying conditions: two concentrations of PdCl₂ (100 and 200 mM), two pH levels (2.0 and 12.0), and before/after cationization using DADMAC. SEM and EDX analyses confirmed the deposition and elemental composition of nanoparticles. Particle size before cationization ranged from 7.8 ± 2.5 to 11.1 ± 2.5 nm, and 5.5 ± 1.7 to 2.4 ± 0.7 nm. Acidic media and DADMAC treatment is favored for smaller, spherical particles and better dispersion. After PdNPs modification, viscose showed very good – excellent UV-protection (UPF = 35.2–88.0). Excellent antimicrobial activity (microbial reduction = 90.5–94.3%) was obtained for modified viscose against different microbial pathogens. Multi-functional of viscose was prepared with good durability. The data demonstrate that, IR-assisted PdNPs functionalization is an effective and sustainable method for durable multi-performance viscose textiles.

**Supplementary Information:**

The online version contains supplementary material available at 10.1186/s13065-025-01690-0.

## Introduction

Originally, garments were used simply for protection and modesty. However, with evolving consumer lifestyles and rising expectations, there is a growing demand for textiles that offer more than just basic utility. Modern consumers increasingly seek garments with advanced functionalities, such as protection from ultraviolet (UV) radiation, antibacterial properties, and resistance to water, stains, and pests [1–3]. These requirements have shifted textile development from basic production to sophisticated functionalization often involve the integration of nanomaterials and chemical treatments. Among the wide range of fibers used in textiles, viscose as regenerated cellulose fiber is valuable for its softness and biodegradability. However, it lacks inherent performance features like UV resistance and antimicrobial activity. To address these limitations, textile researchers have increasingly explored the application of functional agents and nanotechnology [4–6]. Yet, traditional finishing methods often rely on wet chemical processes that consume large volumes of water, energy, and synthetic chemicals, contributing to environmental degradation [7]. As a sustainable alternative, infrared (IR) radiation has emerged as an efficient tool for textile treatment. IR-assisted method allows for rapid and energy-efficient/in-situ nanoparticle synthesis directly on fabric surfaces. In this study, IR technology was employed to immobilize palladium nanoparticles (PdNPs) onto viscose fabrics, aiming to enhance their durability, UV protection, and antimicrobial efficacy while minimizing environmental impact [6, 8, 9].

The integration of nanotechnology into the textile sector has sparked significant global interest due to its potential to revolutionize the performance and functionality of both natural and synthetic fibers [10–14]. Nanotechnology refers to the manipulation and control of materials at the nanoscale, typically between 1 and 100 nanometers, where unique physical and chemical properties emerge [15, 16]. At this scale, materials can exhibit novel optical, mechanical, thermal, and antimicrobial properties that differ significantly from their bulk counterparts. This capability allows scientists to design and tailor fibers with enhancing the incorporation of nanoparticles (NPs) directly with textile surfaces. Through this process, nanotechnology enables the development of advanced textile materials with improved abrasion resistance, breathability, durability, and added functionalities such as UV protection, antimicrobial action, self-cleaning surfaces, and water or stain repellency [5, 17, 18]. Unlike conventional finishing techniques, nanotechnology-based processes often use fewer chemicals and less water, making them more sustainable and energy-efficient.

By integrating nanomaterials into these fibers, manufacturers aim to combine the best qualities of both categories to enhance the performance without sacrificing comfort or sustainability [19–21]. The early 2000 s marked a turning point in textile innovation, as commercial applications of nanotechnology began to emerge. Metal-based nanoparticles such as silver, gold, copper oxide, zinc oxide, and titanium dioxide were applied to textiles to confer multifunctional properties like UV resistance, antimicrobial effects, and stain repellency [22–24]. As the field progresses, nanotechnology continues to offer promising avenues for developing high-performance, multifunctional fabrics suitable for apparel, technical textiles, and healthcare applications [25]. Palladium (Pd) is one of the six platinum group metals (PGMs), distinguished by its unique physical and chemical properties. With an atomic number of 46, it is the least dense and has the lowest melting point among PGMs, making it particularly versatile in industrial applications [26, 27]. When applied to fabrics, PdNPs can impart desirable characteristics such as antibacterial activity, UV protection, thermal resistance, and enhanced durability. Their incorporation transforms ordinary fabrics into smart and multifunctional materials suitable for use in medical textiles, sportswear, protective clothing, and daily wear [28, 29].

IR-assisted/in-situ nucleation of nanoparticles likely demonstrates very low energy consumption (localized, rapid heating) and strong adhesion of NPs within textile matrix, attributing to direct nucleation at the substrate interface. While microwave and laser methodologies provide helpful analogies, representing fast synthetic technique of well-dispersed PdNPs, but they’re not less efficient compared to infra-red assisted immobilization [30, 31].

In the present study, as the infra-red assisted technique for controllable nucleation of PdNPs was not previously investigated. Whereas, such technique can be expressed as ecofriendly, sustainable and efficient technique. Therefore, the current study was interestingly considered with the fabrication of antimicrobial and UV-protective viscose textiles via in-situ deposition of PdNPs. Herein, the immobilization process was performed under both acidic and alkaline conditions, and the influence of fabric cationization on efficiency of immobilization for PdNPs, was thoroughly examined. The size distribution of PdNPs in the supernatant was assessed using transmission electron microscopy (TEM). To characterize the surface morphology and elemental composition of PdNPs-modified textiles, a combination of analytical techniques was employed, including scanning electron microscopy (SEM), Fourier-transform infrared spectroscopy (FTIR), X-ray diffraction (XRD), and X-ray photoelectron spectroscopy (XPS). The functional performance of the treated viscose fabrics was evaluated by examining their colorimetric, ultraviolet (UV) protection, and antimicrobial activity. Furthermore, the durability of the imparted functionalities was assessed through repetitive laundering cycles, to confirm the stability and wash-fastness of the modified properties.

## Materials and experiments

### Materials and chemicals

All chemicals used in this study were of analytical grade and used without further purification. Palladium(II) chloride (PdCl₂, 99%), sodium hydroxide (NaOH, 99%), sodium carbonate (Na₂CO₃, ≥ 99.5%), and glacial acetic acid (CH₃COOH, ≥ 99.7%) were obtained from Sigma-Aldrich. Diallyl dimethyl ammonium chloride (DADMAC, C₈H₁₆NCl, ≥ 97%) was supplied by Sigma-Aldrich and used as a cationic agent for fiber modification. A commercially available non-ionic surfactant was employed as received to enhance dispersion and wetting properties during treatment processes. Bleached plain weave viscose fabric was supplied by El-Nasr Company for Spinning, Weaving, and Dyeing (El-Mahallah El-Kubra, Egypt). The fabric was used as the base substrate for nanoparticle immobilization without any further chemical treatment.

### Procedure

#### Viscose cationization

According to literature, the surface decoration of viscose can be successively modified via cationization, wherein hydroxyl groups of cellulose interact with cationizing agent, to enhance the fiber’s affinity to bind with negatively charged species or nanoparticles (Li, Zhai et al. 2022; Negi 2024). In this study, di-allyl dimethyl ammonium chloride (DADMAC) was used as the cationizing agent. A molar ratio of 1:2 (DADMAC: NaOH) was applied to prepare the cationization solution. The viscose fabric samples were prepared using a pad-dry-cure methodology, where the fabric was soaked within DADMAC–NaOH mixture through two dips and two nips, then passed through rollers for wet pick-up of approximately 100% [32]. The prepared samples were subsequently dried at 80 °C for 10 min and then cured at 130 °C for 3 min. Finally, in the curing step, the fabrics—identified as non-cationic (Vs) and cationic viscose (Q-Vs) were thoroughly rinsed with tap water and subsequently with 1% (v/v) acetic acid for neutralization. The samples were then subjected for repeated washing cycles to ensure the removal of unreacted reactants and dried again at 80 °C [33]. This chemical modification demonstrates the quaternary ammonium groups to the cellulose backbone, to change its surface polarity and in turn to improve the interaction with various functional additives such as metal nanoparticles. These can be reflected in that, the cationic viscose exhibits improved binding affinity, to be particularly applicable for subsequent finishing treatment.

#### In situ clustering of PdNPs within viscose

PdNPs can be ascribed a safer and more sustainable alternative for the conventional hazardous chemical reagents, due to their potentiality for nucleation through environmentally friendly methods, such as infrared (IR)-assisted method. In this approach, PdNPs were in-situ immobilized onto both cationic and non-cationic viscose fabrics under the effect of infrared (IR) radiation. For each trial, 1.0 g of fabric was put in IR equipment-compatible cups, along with an appropriate volume of distilled H_2_O. 100 mg/L and 200 mg/L of palladium chloride (PdCl₂) solutions were added, and the pH was adjusted to either 2.0 (acidic) or 12.5 (alkaline), respectively, as referred in Table 1. The reaction liquors were exposed to infrared dyeing machine at 100 °C for 1 h. At the end of the reaction duration, the treated samples were carefully removed, rinsed thoroughly with tap H_2_O, and dried at 70 °C. The samples were then kept for subsequent instrumental analyses, including morphology, elemental distribution, and evaluation of their functional characters.

### Characterization and instrumental analysis

The morphological characteristics of the treated viscose fabrics were analysed using a high-resolution scanning electron microscope (HRSEM, Quanta FEG 250, FEI Company, Netherlands). Additionally, the shape and size distribution of PdNPs in the remaining colloidal supernatant were evaluated using a high-resolution transmission electron microscope (HR-TEM, JEOL-JEM-1200, Japan), assisted by 4 pi image analysis software (USA). Elemental analysis was conducted via energy-dispersive X-ray spectroscopy (EDX, AMETEK EDAX system). To identify surface functional groups and assess chemical changes following PdNPs immobilization, Fourier-transform infrared (FTIR) spectroscopy was performed using a JASCO FT/IR 6100 spectrometer. Scanning was conducted at 2 cm⁻¹ resolution, with 32 scans at a scanning speed of 2 mm/s. Crystallinity and phase analysis were determined by powder X-ray diffraction (XRD) using a Philips X’Pert MPD diffractometer operated at 40 kV and 50 mA, employing Cu Kα radiation (λ = 1.5406 Å) at room temperature. The oxidation state and surface composition of palladium in the modified textiles were analyzed using X-ray photoelectron spectroscopy (XPS, Kratos Ultra system**)**, where samples were exposed to Al Kα radiation (1486.6 eV) under ultra-high vacuum.

**Table 1:** Preparation details and color data for the functionalized viscose fabrics by Pd

Fabric	Sample	PdCL_2_ (mL, 1 mM)	pH ^a^	L*	a*	b*	YI E313 [D65/10]
Viscose	Vs	–	–	86.52 ± 1.14	0.06 ± 0.13	0.48 ± 0.22	5.22 ± 1.33
	Vs-IR-1	100	12.0	72.49 ± 2.03	2.48 ± 0.52	28.18 ± 2.10	58.93 ± 2.75
	Vs-IR-2	200	12.0	63.23 ± 1.98	4.94 ± 1.02	28.45 ± 1.84	67.57 ± 2.18
	Vs-IR-3	100	2.0	76.21 ± 1.75	−0.44 ± 0.33	17.62 ± 1.53	33.05 ± 2.01
	Vs-IR-4	200	2.0	70.35 ± 1.47	3.64 ± 0.51	33.29 ± 2.47	66.23 ± 3.10
Cationized Viscose	Q-Vs	–	–	78.6 ± 2.01	0.41 ± 0.07	6.01 ± 1.05	13.5 ± 1.24
	Q-Vs-IR-1	100	12.0	68.96 ± 1.23	2.61 ± 0.27	27.46 ± 2.16	59.83 ± 2.41
	Q-Vs-IR-2	200	12.0	59.45 ± 1.46	5.31 ± 0.67	28.12 ± 1.83	70.08 ± 2.88
	Q-Vs-IR-3	100	2.0	72.93 ± 1.88	1.94 ± 0.45	25.38 ± 1.92	53.56 ± 2.06
	Q-Vs-IR-4	200	2.0	68.03 ± 1.59	3.27 ± 0.69	27.65 ± 2.24	61.37 ± 2.45

The reaction is performed for at 100 °C for 1 h

^a^pH is adjusted by NaOH (1 N)

Colorimetric properties of treated fabrics—such as absorbance, color strength (K/S), lightness (L), chromatic coordinates (a, b*), and whiteness index were all assessed using an Ultra Scan Pro spectrophotometer (Hunter Lab, USA) equipped with a pulsed xenon light source. L* values represent lightness (0 = black, 100 = white), a* indicates the red–green axis, and b* reflects the yellow–blue axis [34, 35]. Measurements were conducted in duplicate to calculate average values. Tensile strength and elongation at break as mechanical properties, were measured for viscose fabrics before and after treatment with PdNPs by Asano machine MFG Co – Japan according to the standardized method of ASTM D2256 − 66 T [36]. For each sample, two measurements were carried out and the mean values were only recorded. Ultraviolet protection efficiency was examined using a JASCO V-750 UV–Vis spectrophotometer (Japan). Transmission spectra (T%) were recorded over UV-A (315–400 nm) and UV-B (280–315 nm) ranges. The ultraviolet protection factor (UPF) was calculated according to AATCC Test Method 183-2010. Each measurement was performed in duplicate, and the mean values with standard deviations were reported.

The antimicrobial efficacy of all treated viscose fabrics, both before and after repeated washing cycles, was assessed against three pathogenic microorganisms using the standard quantitative method [37, 38]. The tested strains included: *Escherichia coli* ATCC-25,922 (Gram-negative bacteria), *Staphylococcus aureus* ATCC-6538 (Gram-positive bacteria), and *Candida albicans* ATCC-10,231 (fungus). Each microbial strain was cultured in a suitable broth medium and adjusted to a standardized suspension. 100 µL of the microbial suspension was evenly spread over nutrient agar plates. Subsequently, square samples of the modified and control fabrics were carefully placed onto the inoculated agar surfaces. The plates were then incubated at 37 °C for 24 h under shaking conditions to ensure optimal microbial growth and fabric-microbe interaction. Following incubation, microbial growth was assessed via spectrophotometer. The absorbance of the culture medium was measured at 550 nm using a JASCO UV 630 spectrophotometer. Antimicrobial activity was calculated by determining the percentage reduction in microbial growth compared to untreated control samples. This evaluation provided insight into the effectiveness of palladium nanoparticle-treated fabrics in inhibiting bacterial and fungal proliferation, both in their freshly modified and washed states, to reflect the durability and robustness of the antimicrobial functionality.

The durability of the treated viscose fabrics against the repetitive washings was performed according to the standardized method concerning the home launder washing test of AATCC 2008 [39]. The washing process was carried out via immersing the fabrics in a solution (with material to liquor ration of 1:100) containing 2 g/L sodium carbonate and 1 g/L non-ionic detergent at 50 °C for 15 min under continuous stirring. Consequently, fabrics were taken out, rinsed with tap water, and dried at 70 °C. The washing process was repeated 10 times to achieve 10 consecutive washing cycles.

## Results and discussion

### In-situ clustering of PdNPs

**Fig. 1:**
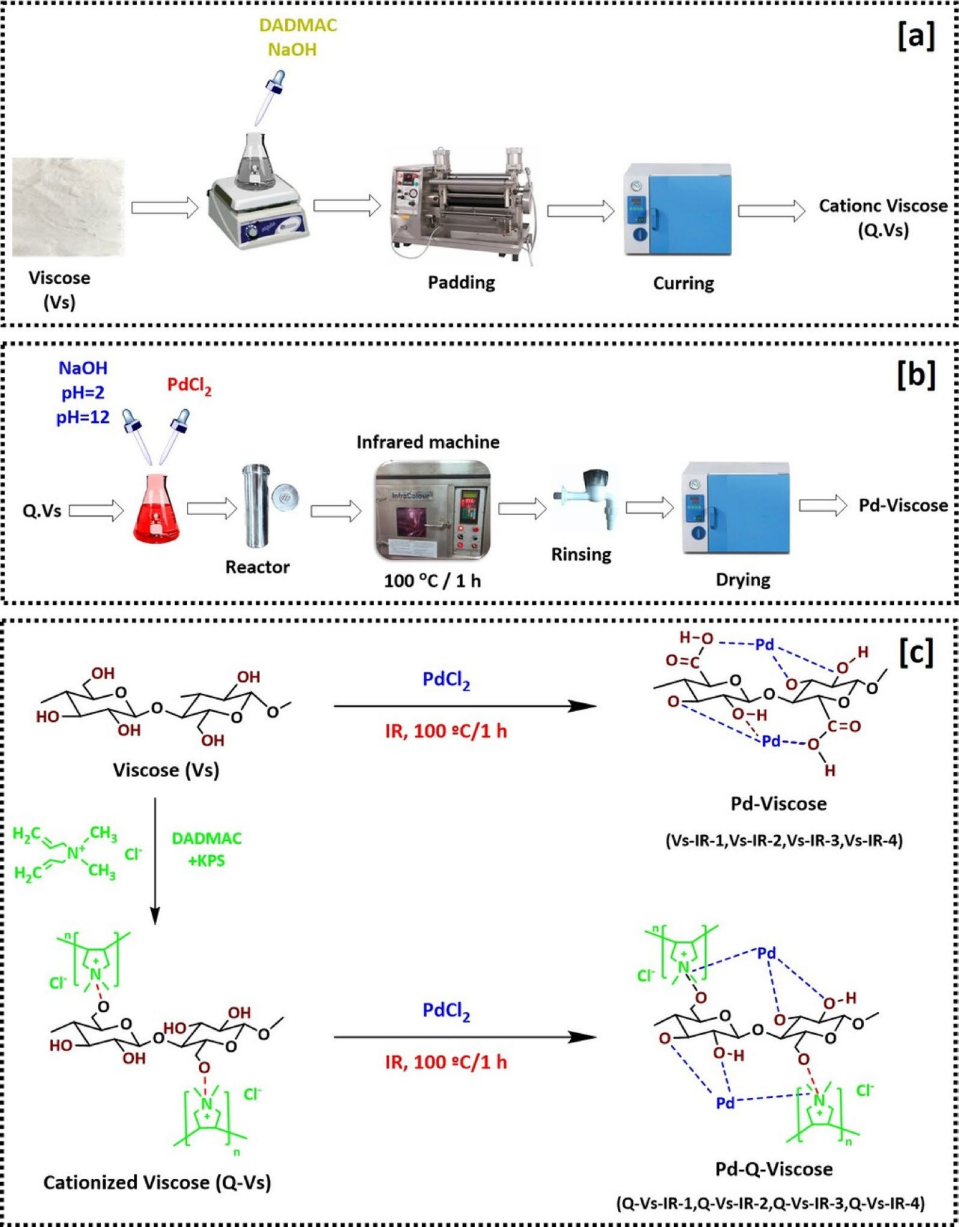
Schematic diagram for; **a** cationization process, **b** modification of viscose fabrics with PdNPs and **c** reaction mechanism between Pd and viscose/cationic viscose

According to the literature, only few studies were interested in the self-implantation of NPs within viscose fabrics to achieve durable antimicrobial and UV-protection performance under the effect of infrared (IR) irradiation. This research emphasizes the significance of nano-palladium for imparting the multifunctional finishing to viscose textiles. To activate the viscose fabrics, cationization process of viscose was performed via interaction with DADMAC as presented in Fig. 1a. Secondly, the process for incorporation of PdNPs within the matrix was schematic in Fig. 1b.

The mechanism of in-situ clustering for PdNPs within the viscose matrix can be hypothesized as a result of a redox reaction between palladium ions and the terminal functional groups of the viscose polymer chains [40, 41]. To enhance the interaction between the viscose matrix and PdNPs, cationization of viscose is performed for chemical modification of hydroxyl groups using di-allyl dimethyl ammonium chloride (DADMAC). As the reaction proceeds, the hydroxyl groups of cellulose are polarized, introducing cationic charges onto the fabric surface. This modification changes the intrinsic characters of cellulose, to promote more effective interaction with palladium nanoparticles and thereby improving the fabric’s functional potency. The direct stabilization of PdNPs was systematically investigated in relation to its impact on the antimicrobial action, UV-protective performance, and coloration of viscose fabrics. The proposed interaction mechanism between PdNPs and the modified viscose fabrics is illustrated in Fig. 1c.

### TEM micrographs and size distribution

**Fig. 2:**
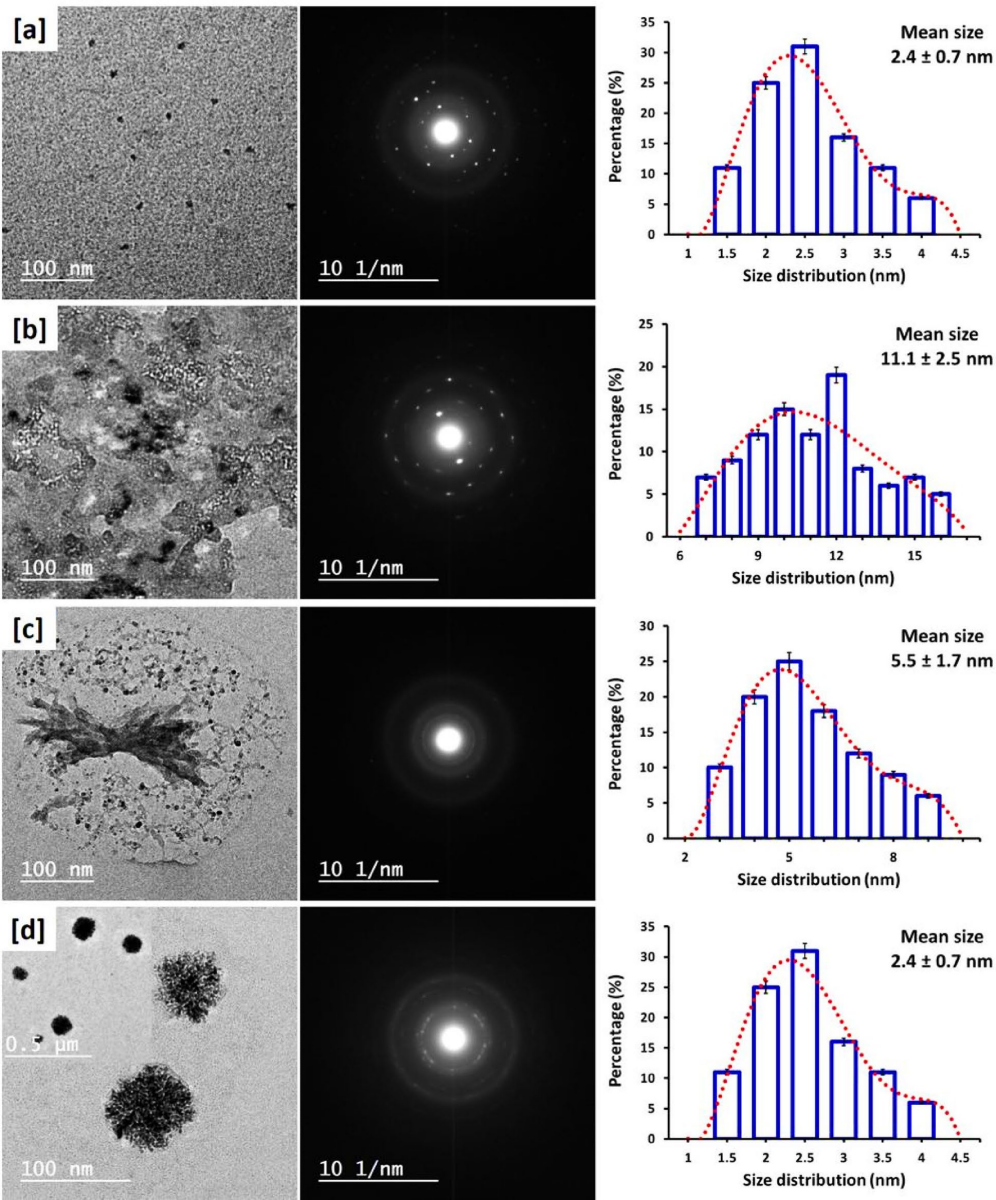
TEM micro-images, diffraction and size distribution for PdNPs in the residual solutions; **a** Vs-IR-2, **b** Vs-IR-4, **c** Q-Vs-IR-2, **d** Q-Vs-IR-4

Transmission electron microscopic images (TEM) were collected to investigate the morphology, dispersity, and particle size distribution of the in situ synthesized palladium nanoparticles (PdNPs). Figure 2 represents TEM micrographs, selected area electron diffraction (SAED) patterns, and particle size distribution profiles of PdNPs ingrained under different synthesis conditions: (Fig. 2a & b) within unmodified viscose fabrics using 200 ppm palladium chloride in alkaline and acidic media, respectively, and (Fig. 2c, d) within cationic viscose fabrics under the same palladium concentration and pH conditions. All PdNPs were synthesized under IR irradiation [42, 43]. TEM images reveal the controlled nucleation of well-dispersed, spherically shaped PdNPs. In Fig. 2a and b, PdNPs were in situ nucleated within viscose fabrics. The nanoparticles were characterized with the polycrystalline structure, as affirmed by SAED patterns. This structural and morphological out coming is attributed to the dual role of the hydroxyl (–OH) groups in viscose: they act both as reducing agents, facilitating the conversion of Pd²⁺ ions to metallic Pd⁰, and as stabilizers, to inhibit the agglomeration of NPs. In alkaline conditions, the hydroxyl groups are more reactive, resulting in smaller particle size (7.8 ± 2.5 nm) compared to those clustered in acidic medium (11.1 ± 2.5 nm). In Fig. 2c and d, PdNPs were nucleated within cationic viscose fabrics. The existence of cationic groups from DADMAC contributed to enhance the stabilization of the nanoparticles, resulting in further reduction for the particle size. The average size was 5.5 ± 1.7 nm in alkaline media and 2.4 ± 0.7 nm in acidic media. The smallest nanoparticles were observed in acidic conditions, suggesting the enhanced stabilization due to the protonation and activation of the cationic DADMAC groups [44, 45].

### SEM images and EDX data

**Fig. 3:**
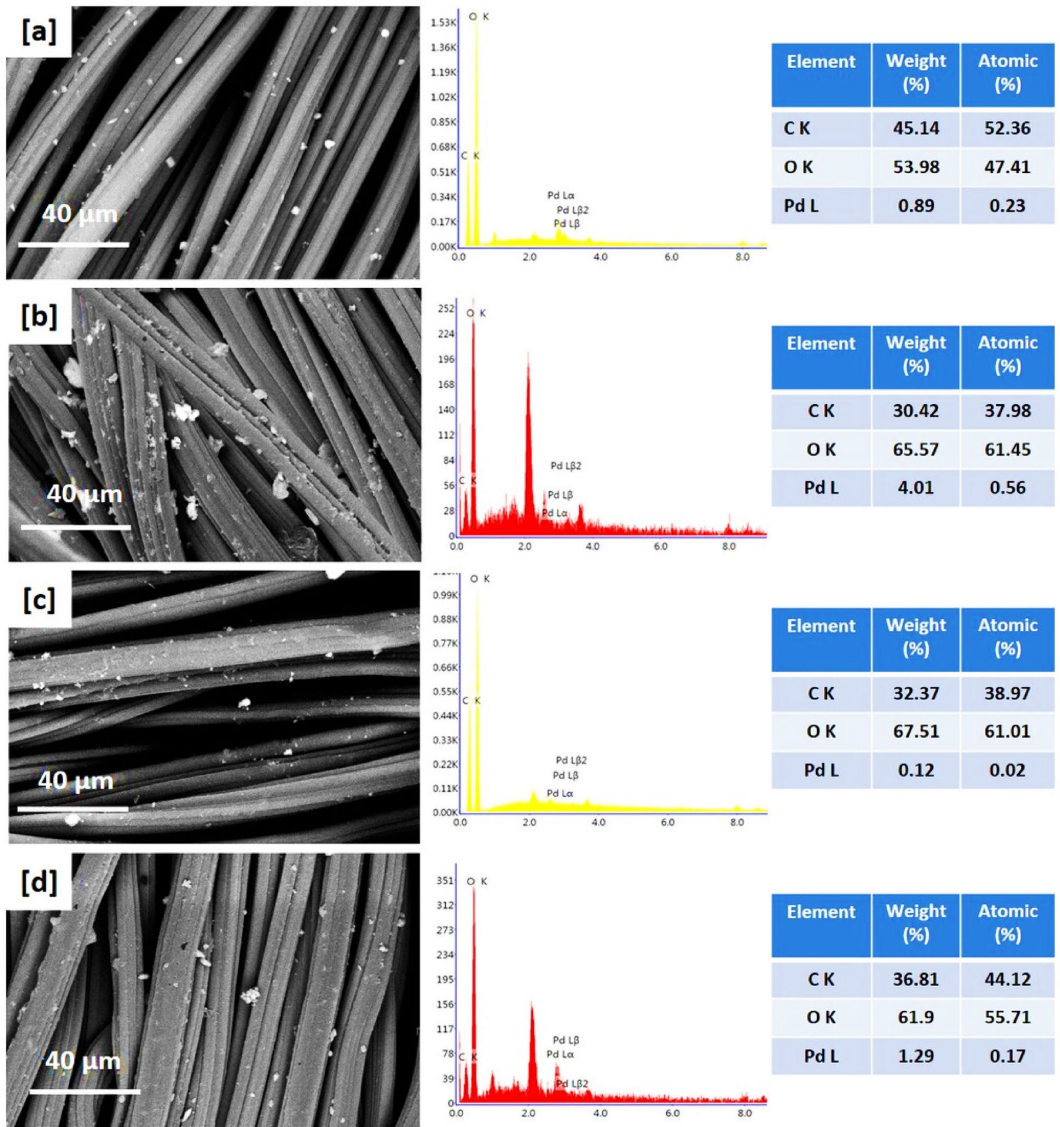

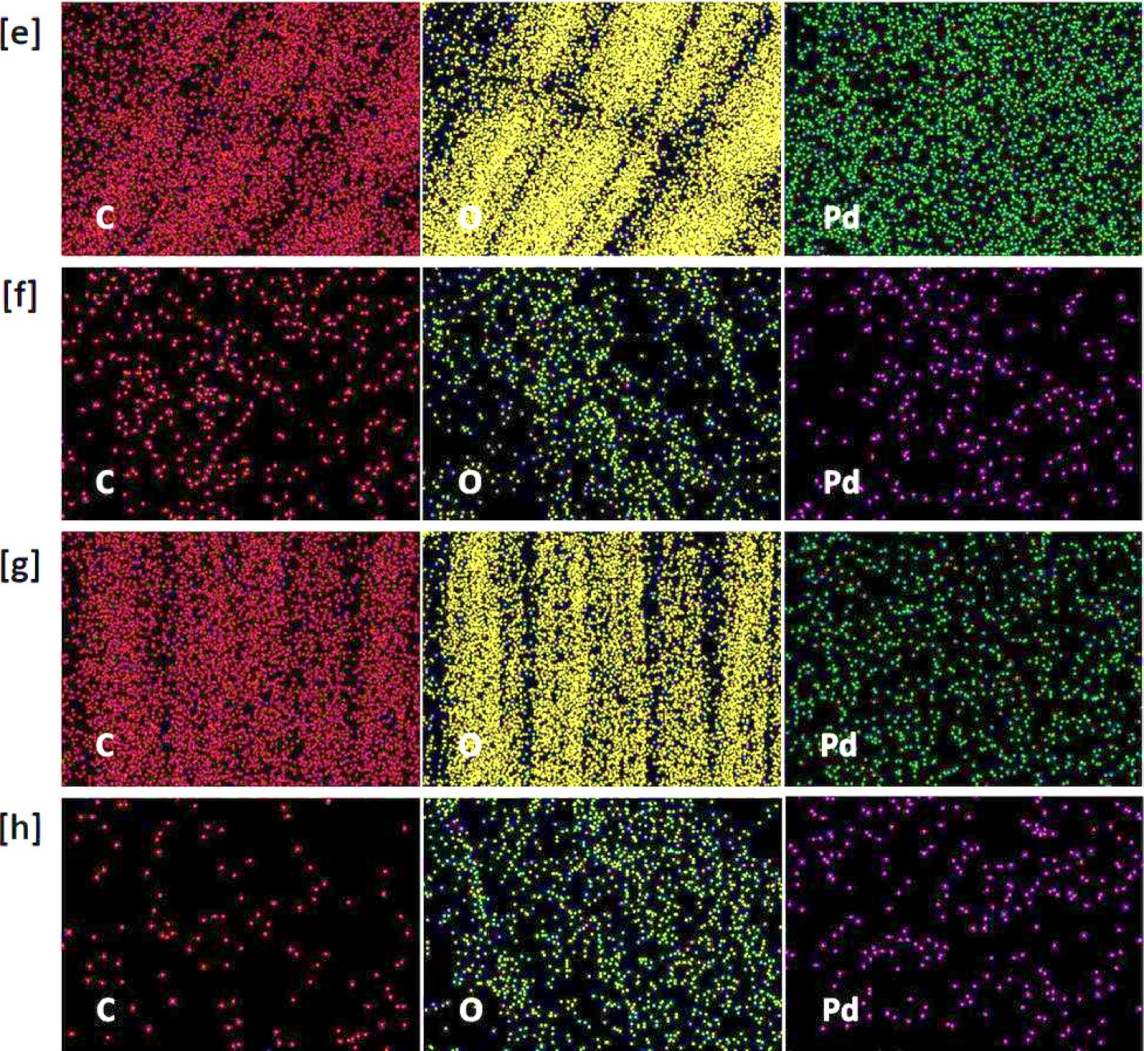
Scanning micro-images, EDX analysis and mapping for the functionalized viscose fabrics; **a**,** e** Vs-IR-1, **b**,** f** Vs-IR-2; **c**,** g** Vs-IR-3 and **d**,** h** Vs-IR-4

**Fig. 4:**
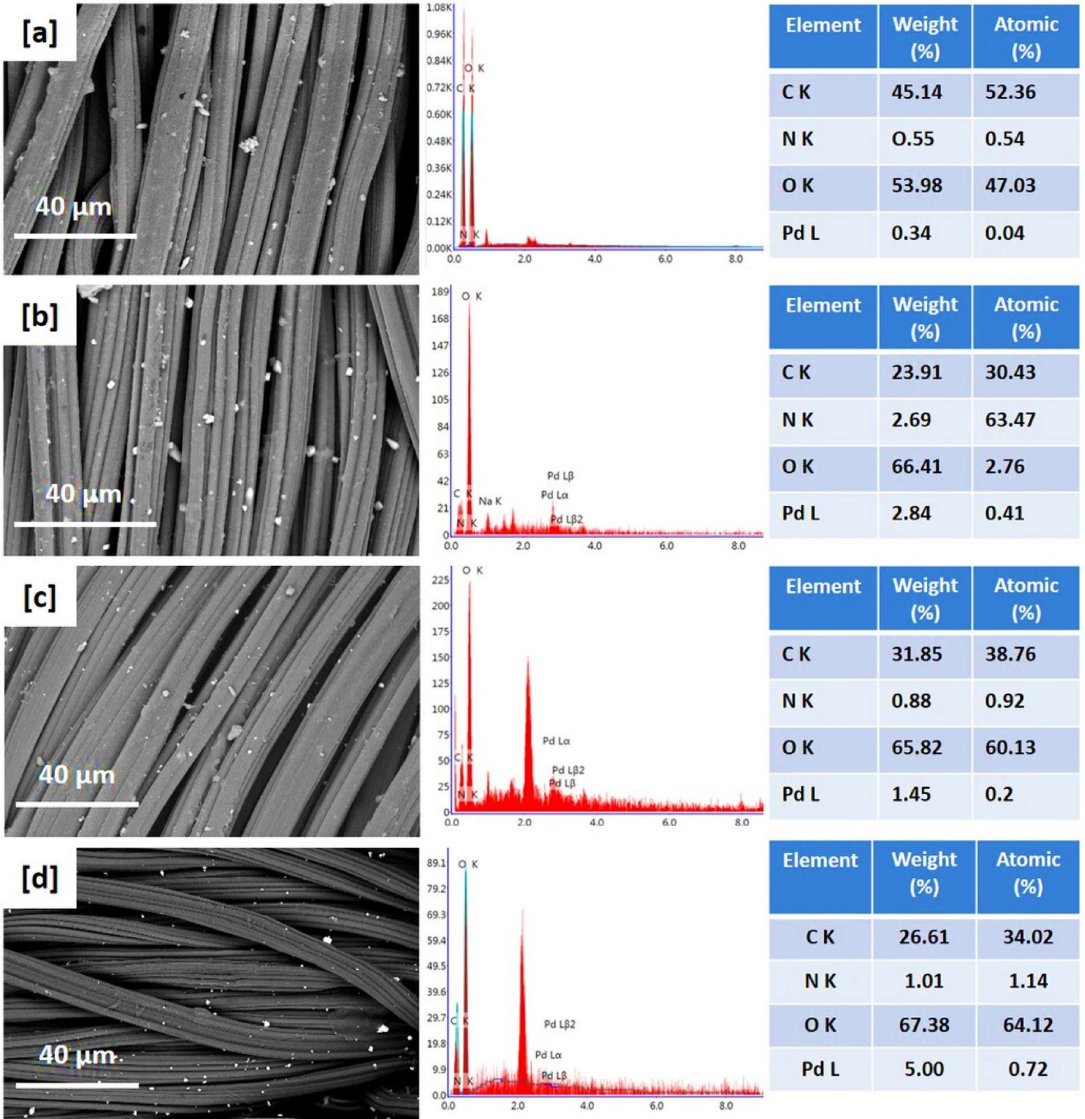

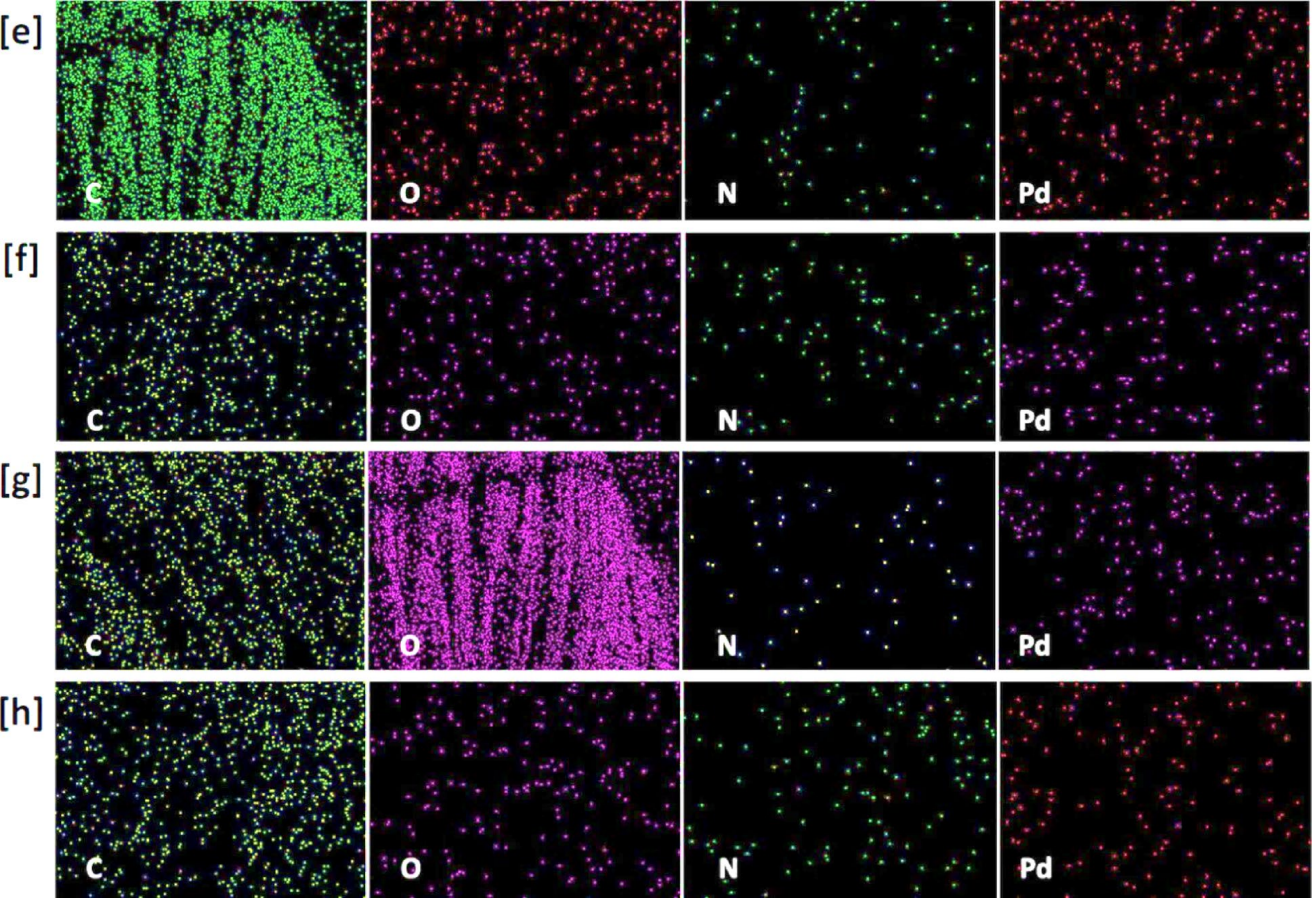
Scanning micro-images, EDX analysis and mapping for the functionalized cationic viscose fabrics; **a**,** e** Q-Vs-IR-2, **b**,** f** Q**-**Vs-IR-4; **c**,** g** Q-Vs-IR-3 and **d**,** h** Q-Vs-IR-4

Scanning Electron Microscopy (SEM) was analysed to examine the surface morphology of viscose fibres via the in-situ nucleation of PdNPs, as depicted in Figs. 3 and 4. SEM images reveal that PdNPs were appeared as small spherical particles, homogeneously distributed on the fibre surface and within the interstitial spaces of the textile matrix [46, 47]. At higher precursor concentration (200 ppm), agglomeration of nanoparticles was observed, resulting in the appearance of larger aggregates. Under acidic conditions (Fig. 3c and d), PdNPs were noted with smaller size compared to those synthesized in alkaline media (Fig. 3a and b), attributing to the higher activation of quaternary ammonium groups from DADMAC, which promote the nucleation of PdNPs and stabilization [48, 49]. These observations are consistent with TEM findings.

The presence and distribution of palladium on the viscose fabrics were further affirmed by EDX. The palladium content was approximately 0.89% and 0.12% at precursor concentration of 100 ppm, increasing to approximately 4.01% and 1.29% at 200 ppm in alkaline and acidic conditions, respectively. EDX mapping indicated uniform distribution of PdNPs across the fabric surfaces, confirming the SEM observations. Moreover, the Pd particles were obviously seen over the cationic viscose fibres after 5 and 10 repetitive washing cycles (Supplementary file, Figure S1). From EDX, Pd signals were still observed after washing and the Pd content was reduced to be 0.14 after 10 washings, which reflect the good durability of the modified fabrics against washing.

Recent studies were demonstrated the efficacy of advanced imaging and analytical methods for the characterization of the morphology and distribution of nanoparticles. For instance, Nguyen et al. (2024) utilized SEM imaging combined with deep learning algorithms for accurate identification and analysis of Pd/C nanoparticles, to approve a significant insights into their morphological distribution and structural organization [50]. Similarly, the investigation of the in-situ nucleation of metal nanoparticles (silver, gold, palladium) within cotton/viscose fabrics was performed using SEM and EDX data to affirm the uniform distribution of nanoparticles and the improved functional properties [23, 29, 34, 35]. Furthermore, the studies on the decoration of cellulosic fabrics with silver nanoparticle-based iodide and titanium-organic frameworks have highlighted the significance of uniform distribution of nanoparticles for environmental purposes [51, 52]. These findings underscore the critical role of SEM and EDX analyses in the elucidation of the morphological characteristics and distribution patterns of nanoparticles on the textile substrates.

### FTIR

**Fig. 5:**
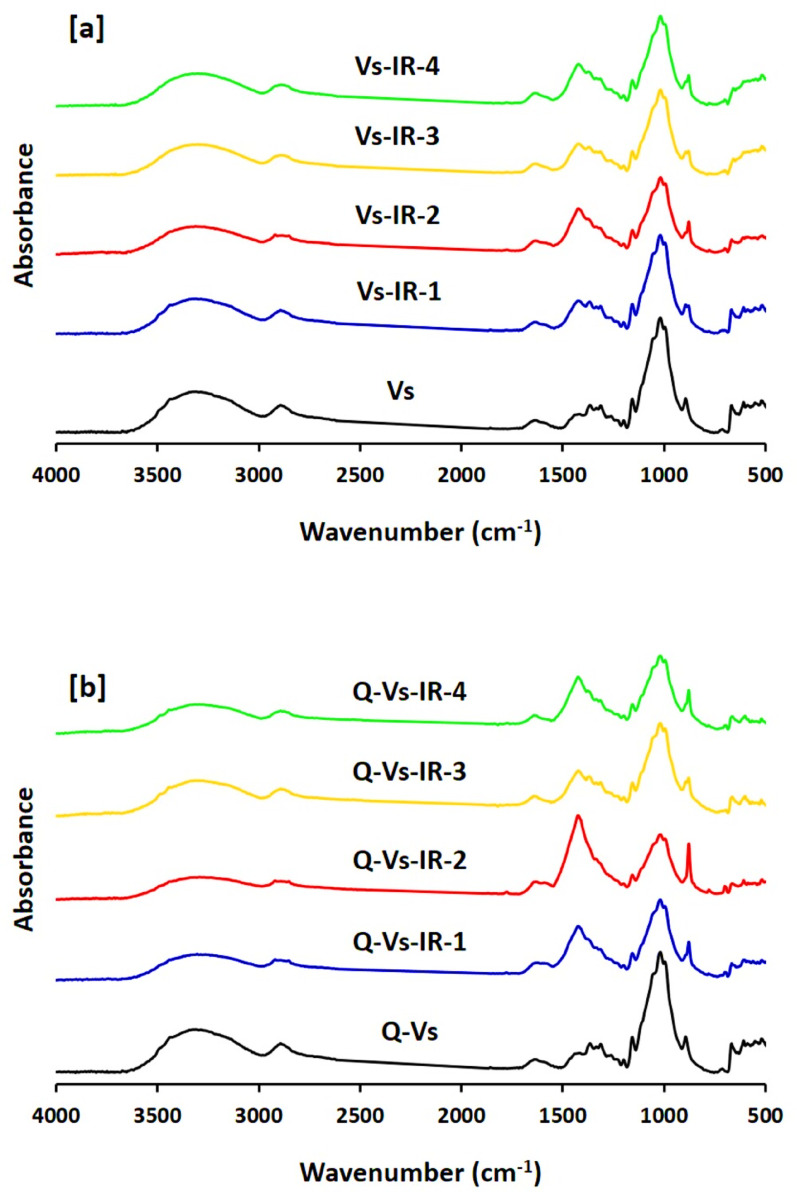
IR spectral results for the functionalized viscose fabrics

The data of Fourier-transform infrared (FTIR) spectroscopy were analysed to examine the chemical composition of the functionalized viscose fabrics before and after the in-situ nucleation of Pd nanoparticles. The absorbance spectra, collected in the wavenumber range of 4000–500 cm⁻¹, are shown in Fig. 5. Figure 5a illustrates the FTIR spectra of pristine viscose fabrics and Pd-treated viscose fabrics, while Fig. 5b shows the corresponding spectra for pristine and Pd-treated cationic viscose fabrics [53, 54]. In Fig. 5a, the characteristic absorption peaks associated with regenerated cellulose (viscose) are evident. A broad peak around 3300 cm⁻¹ corresponds to the stretching vibrations of hydroxyl (–OH) groups and its broadening is attributed to extensive intra- and intermolecular hydrogen bonds. The band at 1640 cm⁻¹ is assigned to overlapping vibrations of carbonyl (C = O) groups and adsorbed water molecules located within the fabric structure. Absorption bands at 2880 cm⁻¹ and 1350 cm⁻¹ are associated with C–H stretching and bending vibrations, respectively, while the peak near 1100 cm⁻¹ is indicative of ether (C–O–C) stretching in the cellulose backbone. Upon in-situ nucleation of palladium nanoparticles under infrared (IR) irradiation, minimal spectral changes were observed. However, slight modification in the intensities of the –OH (3300 cm⁻¹) and C–O–C (1100 cm⁻¹) bands suppose their involvement in the reduction of Pd²⁺ ions and the stabilization (capping) of the nucleated PdNPs [55, 56]. These findings affirm that the core chemical composition of the cellulose remained intact, indicating that the processing conditions, namely acidic or alkaline media and exposing to IR, did not compromise the structural integrity of the viscose matrix.

In the case of cationic viscose fabrics (Fig. 5b), the FTIR spectra revealed no substantial shifts in peak positions or intensities post-treatment, except for a slight decrease in the –OH band intensity. This reduction is correlated to the chemical modification of hydroxyl groups via cationizing with DADMAC, which replaces some –OH groups with quaternary ammonium groups. The retention of other key spectra further supports the stabilization of the cellulose composition during and after PdNPs nucleation. These FTIR findings are consistent with recent studies emphasizing the role of hydroxyl and ether groups in nanoparticles formation and their minimal influence on the structural framework of polysaccharide-based substrates during functionalization [57, 58].

### XRD patterns

**Fig. 6:**
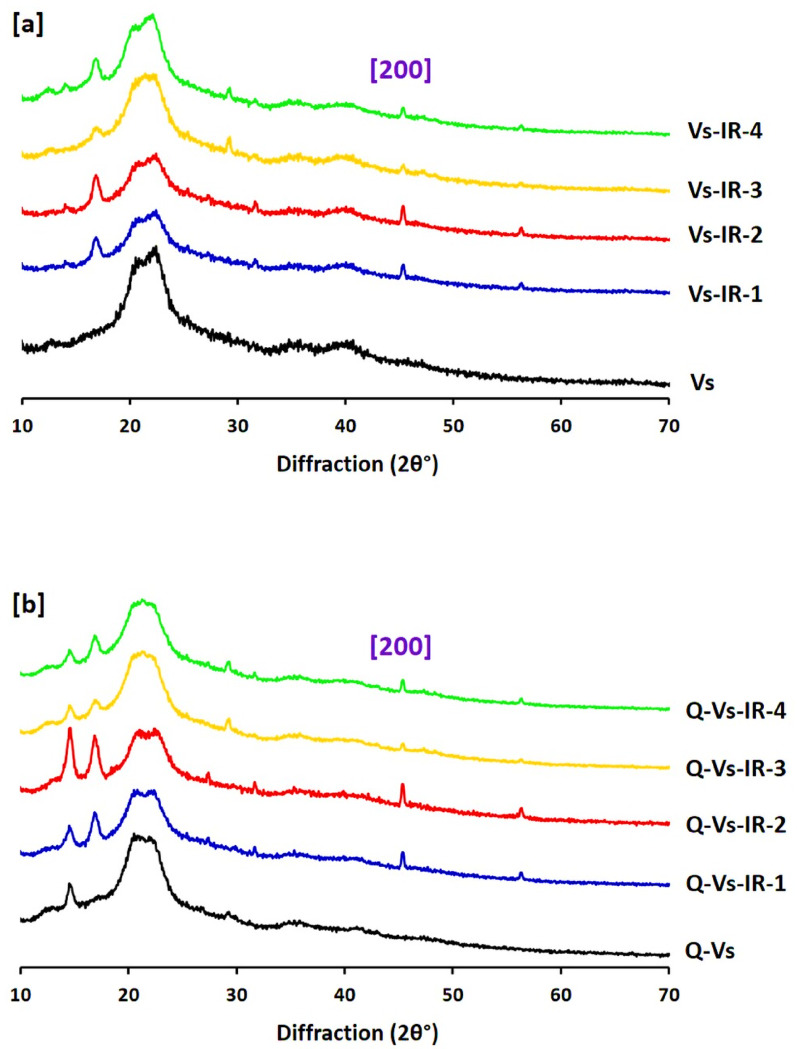
Analysis of XRD for the functionalized viscose fabrics

XRD analysis was performed to investigate the crystallinity of viscose fabrics and to assess the effect of in-situ PdNPs nucleation under different treatment conditions. Viscose crystallinity is primarily determined by the degree of ordered arrangement of cellulose chains, which significantly influences its mechanical stabilization, chemical reactivity, and potential for surface functionalization. Therefore, understanding how Pd treatment affects this property is essential for evaluation of the multi-functionality for the modified viscose fabrics. As shown in Fig. 6a, the XRD pattern of pristine viscose fabrics displays characteristic diffraction peaks at 2θ = 20.5° and 22.5°, corresponding to the (110) and (020) crystallographic planes of cellulose II. These bands reflect the semi-crystalline nature of regenerated cellulose. Following palladium in-situ immobilization under the effect of infrared irradiation, a new diffraction bands appears at 2θ ≈ 47°, which is attributed to the (002) plane of the face-centred cubic (fcc) structure of palladium nanoparticles [59, 60]. This confirms the successful nucleation and crystalline nature of PdNPs within the viscose matrix.

Interestingly, the cellulose diffraction intensity slightly decreased in samples treated under acidic conditions, reflecting a minor disruption or loosening of the cellulose crystalline framework, possibly due to partial acid hydrolysis. In contrast, samples treated in alkaline media retained their original peak intensity, supposing the preservation of crystalline orientation. These observations imply that the nucleation of PdNPs especially under alkaline conditions does not significantly compromise the structural integrity of viscose, a promising indication for maintaining fabric performance after functionalization. For cationic viscose fabrics, both unmodified and PdNPs modified, the XRD profiles (Fig. 6b) were exhibited additional peaks at 2θ ≈ 14.4°, 20.5°, and 22.5**°**, corresponding to the (110), (110), and (020) planes, respectively. These reflections are consistent with the modified cellulose II structure after chemical cationization using DADMAC. The incorporation of PdNPs into cationic viscose led to the same Palladium-related diffraction at 4**7°**, to affirm the crystalline deposition of palladium without significant influence on the original cellulose crystallinity. The lack of notable changes in peak position or intensity suggests that both cationization and PdNPs deposition are compatible with the crystalline structure of viscose, thereby enabling the acquirement of multifunctional fabrics without sacrificing structural fidelity [61, 62]. These findings are in harmony with recent reports indicating that metal nanoparticles immobilization into polysaccharide matrices can be realized without severe crystalline disruption, especially when green or mild synthetic routes such as IR-assisted reduction, are used [23, 63–65].

### XPS analysis

**Fig. 7:**
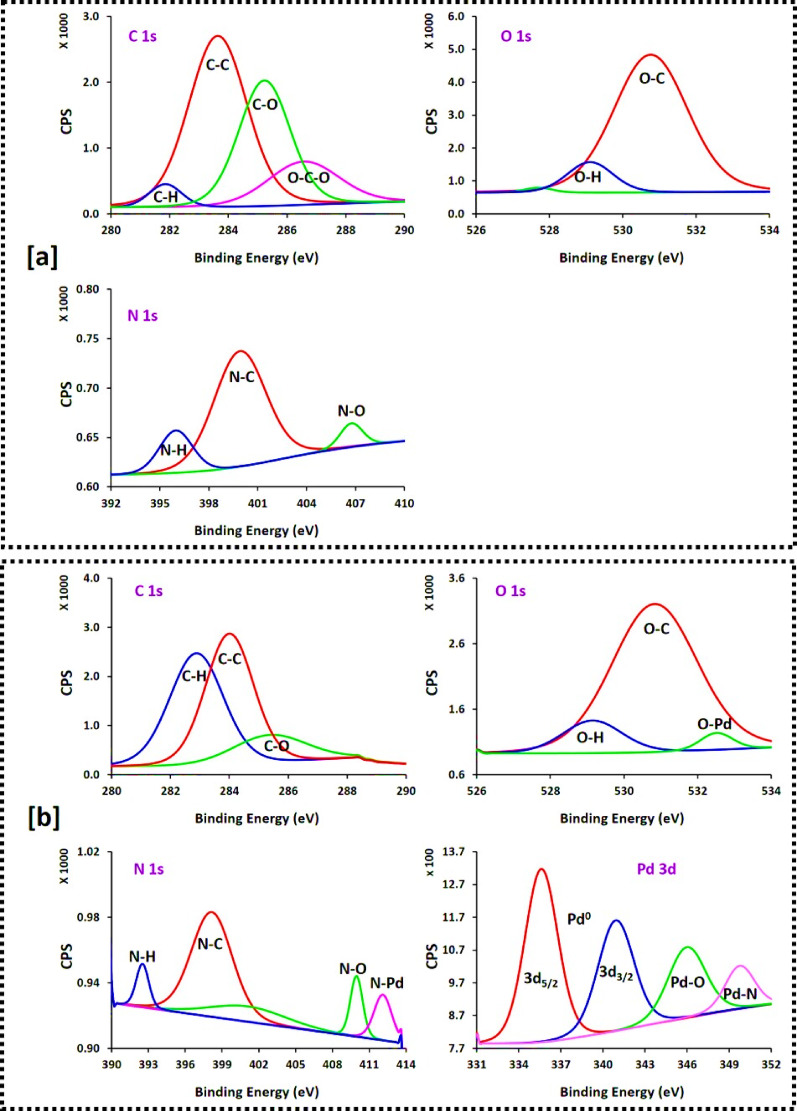
XPS analysis for the functionalized viscose fabric; **a** Q-Vs and **b** Q-Vs-IR-4

XPS was conducted for further confirmation of the chemical composition, oxidation states, and elemental bonding of PdNPs immobilized within the viscose and cationic viscose matrices. XPS used as a sensitive and non-destructive technique for identification of the electronic states of surface-bound elements, especially transition metals like Pd. Figure 7 represents a survey and high-resolution XPS spectra of pristine cationic viscose (Q-Vs) and palladium-functionalized cationic viscose (Q-Vs-IR-4). The survey spectrum of Q-Vs (Fig. 7a) displays core-level bands corresponding to C 1 s, O 1 s, and N 1 s, were observed within the expected binding energy range of 280–288 eV, 528–532 eV, and 392–410 eV, respectively. These peaks are consistent with the presence of cellulose (C and O) and quaternary ammonium groups (N) introduced via DADMAC cationization [66, 67]. The detected peaks for C 1 s are related to the C-H (282.2 eV), C-C (283.6 eV), C-O (285.4 eV) and O-C-O (286.4 eV). Two dominant bands for O 1 s are assigned for O-H (529.2 eV) and O-C (530.8 eV). While N 1 s showed three peaks referring to bonding environments of N-H (396.2 eV), N-C (400.2 eV) and N-O (406.8 eV).

Upon modification with palladium (Q-Vs-IR-4, Fig. 7b), XPS spectra maintained the expected carbon, oxygen, and nitrogen profiles, albeit with minor shifting, attributing to the electronic interactions with palladium. C 1 s deconvolution revealed bands at 282.8 eV (C–H) with higher intense, 284.0 eV (C–C), and 285.6 eV (C–O). While, the bands of O-H (529.2 eV) and O-C (530.8 eV) showed with lower intensity. Shift in N 1 s bands recorded with lower intense for N-C (398.4 eV) and higher intense for N-O (410.2 eV). These bands affirming the stabilization of the cationic quaternary ammonium group and hydroxyl groups in cellulose for the immobilized PdNPs. Moreover, the increasing in the intensity of C-H band is attributed to the redox reaction between cellulose in viscose and Pd^2+^. Most importantly, Pd 3 d region revealed two principal intensive bands at 335.8 eV (Pd 3d₅/₂) and 341.2 eV (Pd 3d₃/₂), which are characteristic for metallic palladium (Pd⁰). This is indicating the successful in situ reduction and immobilization of Pd²⁺ to Pd⁰ within the viscose structure under IR radiation [78]. Furtherly, two low bands for Pd-O (346.2 eV) and Pd-N (350.2 eV) attributing to residual of Pd ions and the coordination interaction between hydroxyl and amine groups and metallic Pd⁰, or it may result from partial re-oxidation of Pd⁰ on the fabric surface due to environmental exposing or surface hydroxyl reactivity [68–70]. Such coexistence of Pd⁰ and Pd–O/Pd-N supports a heterogeneous chemical state distribution of palladium on the fabric surface and affirms its chemical immobilization into the cellulose matrix. These findings are consistent with recent reports on metal–cellulose interactions, where the coordination of PdNPs with cellulose-based supporting template was shown to enhance the NPs dispersion and catalytic stabilization [70, 71].

### Colorimetric analysis

**Fig. 8:**
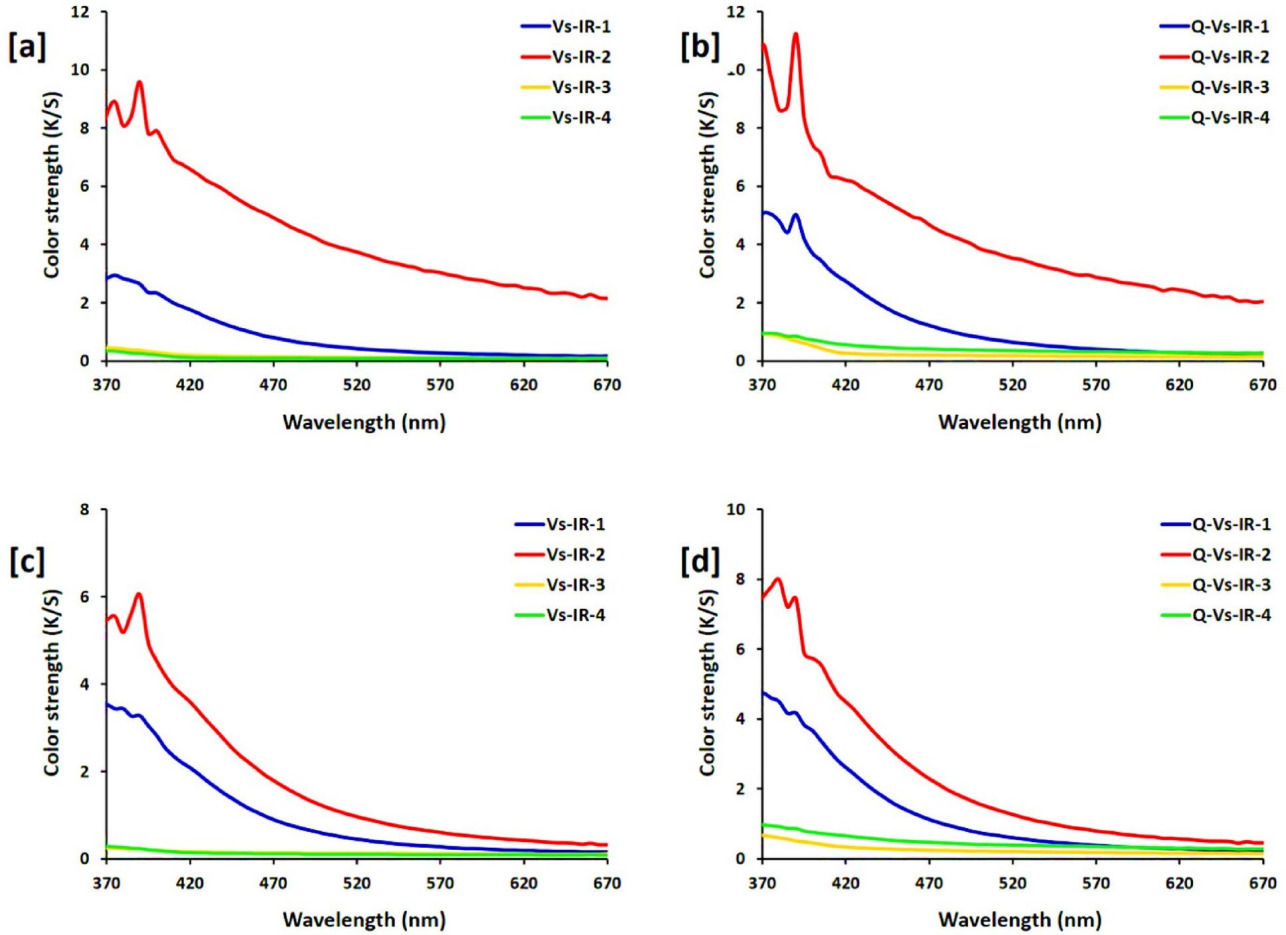
Color properties for the functionalized viscose fabrics; **a**,** b** color strength and **c**,** d** absorbance

The visual transformation of the viscose and cationic viscose fabrics, from white to varying shades of dark yellow, visibly reflects the in-situ nucleation and clustering of PdNPs within the textile matrix. This coloration effects are attributed to SPR phenomena associated with metallic nanoparticles, particularly PdNPs [72, 73]. Colorimetric analysis further quantified these visual observations through K/S (i.e. colour strength), Yellowness Index (YI), and CIELAB colour parameters (L*, a*, b*), as represented in Table 1; Fig. 8. Notably, PdNPs significantly improved the colour strength of both viscose and cationic viscose fabrics, with the highest K/S values were estimated in samples treated with 200 ppm of palladium precursor under alkaline conditions (Vs-IR-2: 67.57; Q-Vs-IR-2: 70.08). These results underscore the effect of both palladium concentration and medium pH, as well as the role of cationization in enhancement of PdNPs absorption and dispersion. The increment in YI, b* (yellow-darkness), and a* (redness), alongside the decrement in L* (lightness), shows the intensified coloration due to successive immobilization of PdNPs, particularly in cationic viscose where ionic interaction that facilitate better stabilization and nucleation of nanoparticles [74, 75].

Further comparison of Pd-viscose and Pd-cationic viscose samples revealed a clear trend in colorimetric performance: Q-Vs-IR-2 ≈ Q-Vs-IR-4 ≫ Q-Vs-IR-1 > Q-Vs-IR-3 and Vs-IR-2 ≫ Vs-IR-4 > Vs-IR-1 > Vs-IR-3, to reveal the synergistic effects of higher palladium concentration and cationic modification. Durability examination after 5 and 10 laundering cycles (Supplementary file, Figure S2 & S3) affirmed the colour fastness of the PdNPs-treated fabrics. The retained K/S, YI, and CIELAB values even after repeated washings reveal the strong interfacial bonding and coordination bonding between PdNPs and cellulose hydroxyl or cationic groups. This suggestion not only reflects the successive nanoparticles stabilization within the matrix but also the potentiality for multi-applications of these functionalized fabrics in real-world applicability, including smart or catalytically active fabrics. Such results reveal the promising applicability of PdNPs for durable and intense textile coloration through sustainable in-situ methodologies.

Recent literature affirms the efficacy of PdNPs for improving the textile coloration and functionalization, aligning with the findings of this approach. For instance, Tang et al. (2020) reported that PdNPs impart strong coloration to cotton fabrics due to surface plasmon resonance (SPR), similar to the yellow-dark hues observed in viscose and cationic viscose fabrics [76]. Additionally, PdNPs-treated fabrics showed superior UV-protection and colour stabilization under alkaline conditions. Fahmy et al. (2020) highlighted the potency of PdNPs for antimicrobial action, to support their multifunctional application in garments [77]. Sarwar et al. (2023) and Kundu et al. (2022) also reported the enhanced colour strength and durability in textiles using Cu₂O and other metallic nanoparticles [78, 79], but PdNPs offered more consistent K/S values and better dispersion. Compared to AgNPs or TiO₂ composites, PdNPs provide greater coloration intensity and washing durability, particularly in cationic viscose due to the enhanced nanoparticle absorption. These comparative results confirm PdNPs’ advantage in delivering both aesthetic and functional textile improvements.

### Mechanical properties

The effect of PdNPs incorporation within the cellulosic polymeric matrix of viscose and cationic viscose on the mechanical properties was investigated through measurement of tensile strength and elongation at break. The obtained results of mechanical properties in Table 2 showed that the mechanical properties for functionalized viscose in basic medium were not significantly changed compared with that functionalized in acidic medium. While the lowering in tensile strength and elongation was 2.7–3.0.7.0% and 5.5–7.3%, respectively for the samples functionalized in alkaline medium (Vs-IR-1, Vs-IR-2). In case of functionalization in acidic medium, higher reduction in tensile strength (9.8–10.9%) and elongation (16.2–17.0%) was observed. The higher reduction in mechanical properties was recorded for cationic samples. The decrement in mechanical properties could be attributed to the chemical medication and the incorporation of PdNPs within the intermolecular matrix of polymeric units in viscose fabrics, results in breaking of the internal bonds. However, the lowering in the mechanical properties of viscose after the PdNPs incorporation is acceptable for the employment in garment production.

**Table 2:** Mechanical properties for the functionalized viscose fabrics

Samples	Tensile strength (MPa)	Elongation (%)
Vs	143.5 ± 2.2	18.5 ± 0.8
Vs-IR-1	142.2 ± 2.5	18.2 ± 0.9
Vs-IR-2	139.6 ± 2.3	17.5 ± 0.7
Vs-IR-3	135.6 ± 2.8	16.7 ± 1.1
Vs-IR-4	129.5 ± 3.0	15.5 ± 1.2
Q-Vs	146.1 ± 2.6	17.7 ± 0.9
Q-Vs-IR-1	143.5 ± 2.2	17.2 ± 0.6
Q-Vs-IR-2	141.7 ± 2.7	6.4 ± 0.9
Q-Vs-IR-3	135.9 ± 2.9	15.3 ± 0.9
Q-Vs-IR-4	130.2 ± 3.1	14.7 ± 1.1

### UV-protection

Nanotechnology represents a technique for the incorporation of UV-blocking agents into fabric matrices through the application of metal nanoparticles such as PdNPs [80]. These nanoparticles possess unique optical characters, to absorbance, reflectance, or scattering harmful ultraviolet (UV) irradiation, thereby improving the UV-protective capabilities of treated fabrics. Owing to their strong absorption in the UV spectrum and high refractive index, PdNPs are especially effective and promisingly hold for application in protective clothing, outdoor gear, and sportswear. In this study, the UV protection performance of both untreated (non-cationic) and cationic viscose fabrics were evaluated using Ultraviolet Protection Factor (UPF) measurements, with the results represented in Table 3. Spectral transmittance results for UV-irradiation (UVR) are provided in **Supplementary file**,** Figure S4**. The UPF value for Pd-treated viscose in alkaline conditions reached 33.7 (classified as “very good”) using of 100 ppm for PdCl₂, which increased to 50.4 (“excellent”) at 200 ppm. Similarly, Pd-modified cationic viscose exhibited superior potentiality, with UPF values of 41.5 and 88.0 (“excellent”) at 100 and 200 ppm, respectively. The acidic medium also yielded considerable protection, with UPF values of 35.2 and 36.5 at the same concentrations. These results emphasize the synergistic role of DADMAC-induced cationization in enhancing PdNPs uptake and distribution across the fabric matrix, thereby boosting the fabric’s UV-blocking ability [81, 82].

**Table 3:** Protection results against ultraviolet radiation for the functionalized viscose fabrics

Samples	UVA T%	UVB T%	UPF	UPF rate
Vs	35.9 ± 1.1	29.8 ± 0.8	3.3 ± 0.6	In sufficient
Vs-IR-1	3.9 ± 0.7	2.7 ± 0.4	33.7 ± 1.2	Very good
Vs-IR-2	2.3 ± 0.6	1.9 ± 0.3	50.4 ± 0.9	Excellent
Vs-IR-3	9.3 ± 0.9	4.7 ± 0.4	16.5 ± 1.4	In sufficient
Vs-IR-4	4.3 ± 0.5	3.0 ± 0.2	29.6 ± 1.6	Good
Q-Vs	19.6 ± 1.0	13.8 ± 0.7	6.8 ± 1.1	In sufficient
Q-Vs-IR-1	3.0 ± 0.5	2.2 ± 0.4	41.5 ± 1.4	Excellent
Q-Vs-IR-2	1.1 ± 0.3	1.1 ± 0.3	88.0 ± 2.2	Excellent
Q-Vs-IR-3	3.3 ± 0.4	2.7 ± 0.5	35.2 ± 1.4	Very good
Q-Vs-IR-4	2.7 ± 0.2	2.8 ± 0.5	36.5 ± 1.7	Very good

To assess the durability of the UV-protective properties, repeated laundering tests were conducted, and the results are displayed in Table 4. After 5 washing cycles, the UPF values of Pd-viscose (Vs-IR-1, Vs-IR-2) dropped to 25.8 and 38.9, corresponding to “good” and “very good” protection levels, respectively. In contrast, Pd-treated cationic viscose (Q-Vs-IR-1, Q-Vs-IR-2) maintained higher performance with UPF values of 38.9 and 66.1 (“very good” and “excellent”). After 10 washing cycles, a more pronounced decrease in UV-blocking ability was observed in Pd-viscose samples, with UPF values reduced to 9.5 (“insufficient”) and 23.5 (“good”). However, Pd-cationic viscose still exhibited durable protection, recording UPF values of 34.2 (“very good”) and 48.0 (“excellent”). These outcomes affirm the robustness of PdNPs nucleation within the viscose matrix, particularly in cationic samples, and validate the use of PdNPs for developing durable, washing fastness, and UV-protective functional fabrics.

Palladium nanoparticles, especially in the nano-scale, characterized with a strong absorption in the deep-ultraviolet ranging (around 200 nm) attributing to the localized surface plasmon resonance (LSPR). This collective oscillation of conduction electron efficiently can absorb the accepted UV photons, to minimize ultraviolet transmittance via the textile matrix [83]. In contrast to zinc oxide or titanium dioxide, which can liberate reactive oxygen species (ROS) under ultraviolet irradiation and risk photo-catalysis, PdNPs primarily are passive absorbers/scattering agents without harmful photo-reactions [84, 85].

The estimated data affirm that the infrared in-situ embedding of PdNPs significantly improved the UV-blocking action of viscose fabrics. Notably, higher UV-protection was observed in samples treated under alkaline conditions and at higher palladium concentrations. The impregnated PdNPs were known to act by reflecting the incident UV-rays, thereby inhibiting its transmission through the fabric and realizing excellent UV-protection [86, 87]. Comparing with with the formerly published reports, the UV-blocking potency of viscose textiles functionalized via in-situ PdNPs immobilization was markedly superior to that of textiles treated with other metallic or nano-metallic absorbers such as silver, copper, or titanium [24, 34, 86, 88–94]. Similar UV-resistance efficiency were also reported in fabrics modified with metal–organic complexes [86, 94], which is further supported the potential of PdNPs as highly effective UV-protective reagents in functional garments.

**Table 4:** Effect of washing on the UV-radiation protection for the functionalized viscose fabrics

	Samples	UVA T%	UVB T%	UPF	UPF rate
5 Washing	Vs-IR-1	4.9 ± 0.4	3.5 ± 0.3	25.8 ± 1.8	Good
	Vs-IR-2	3.2 ± 0.3	.4 ± 0.3	38.9 ± 2.0	Very good
	Vs-IR-3	13.3 ± 1.1	9.6 ± 0.9	9.6 ± 1.2	Insufficient
	Vs-IR-4	5.5 ± 0.6	4.1 ± 0.4	22.1 ± 1.4	Good
	Q-Vs-IR-1	3.2 ± 0.2	2.4 ± 0.2	38.9 ± 1.9	Very good
	Q-Vs-IR-2	1.6 ± 0.2	1.4 ± 0.3	66.1 ± 2.5	Excellent
	Q-Vs-IR-3	4.6 ± 0.3	3.4 ± 0.4	28.1 ± 2.1	Good
	Q-Vs-IR-4	3.9 ± 0.2	2.7 ± 0.3	33.7 ± 1.9	Very good
10 Washing	Vs-IR-1	12.9 ± 0.9	10.0 ± 1.0	9.5 ± 1.3	In sufficient
	Vs-IR-2	5.6 ± 0.6	3.9 ± 0.4	23.0 ± 1.5	Good
	Vs-IR-3	32.0 ± 1.2	27.4 ± 1.2	3.5 ± 0.6	In sufficient
	Vs-IR-4	34.4 ± 1.4	30.5 ± 1.3	3.2 ± 0.8	In sufficient
	Q-Vs-IR-1	3.7 ± 0.3	2.7 ± 0.2	34.2 ± 1.3	Very good
	Q-Vs-IR-2	2.5 ± 0.2	1.9 ± 0.3	48.0 ± 2.2	Excellent
	Q-Vs-IR-3	16.1 ± 1.3	12.0 ± 1.1	8.0 ± 1.6	In sufficient
	Q-Vs-IR-4	14.0 ± 1.1	10.1 ± 1.2	9.3 ± 1.4	In sufficient

### Antimicrobial performance

**Fig. 9:**
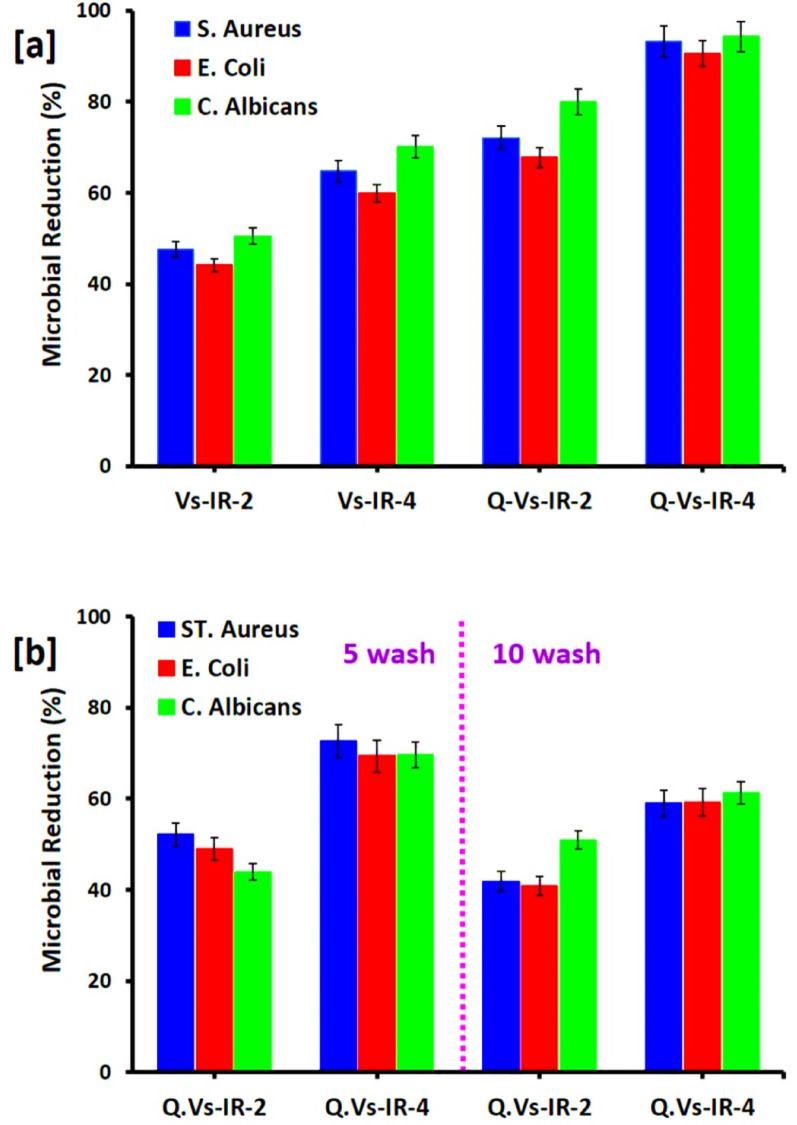
Biological activities for the functionalized viscose fabrics; **a** before washing and **b** after washing

The pivotal role of PdNPs in functionalization viscose fabrics for antimicrobial purpose was studied. The impact of PdNPs’ in-situ immobilization on the biological activities of the fabrics was assessed by evaluation of microbial growth reduction percentages against the examined pathogenic strains; *Staphylococcus aureus*, *Escherichia coli*, and *Candida albicans*, as represented in Fig. 9 [26, 95]. The antimicrobial potency of the modified fabrics was significantly improved with increment palladium concentrations under both acidic and basic conditions. Notably, cationization prior to incorporation of PdNPs further amplified this antimicrobial action [96, 97]. For instance, viscose fabrics treated with 200 ppm Pd in alkaline (Vs-IR-2) and acidic (Vs-IR-4) media exhibited microbial reduction values of 47.5%, 44.1%, and 50.5%, and 64.8%, 59.9%, and 70.1%, respectively, against *S. aureus*, *E. coli*, and *C. albicans*. Upon cationization, these values increased substantially to 72.1%, 67.8%, and 79.9% for Q-Vs-IR-2, and 93.2%, 90.5%, and 94.3% for Q-Vs-IR-4, approving the superior antimicrobial performance attributed to improved PdNPs immobilization.

To evaluate durability, the antimicrobial performance of Pd-modified cationic viscose fabrics was also examined after 5 and 10 washing cycles (Fig. 9b). Remarkably, Q-Vs-IR-2 retained significant activity post-washing, with reduction % of 52.1%, 49.0%, and 43.9% after 5 washing cycles, and 41.9%, 40.8%, and 50.9% after 10 washing cycles against the respective microbial strains. Similarly, Q-Vs-IR-4 demonstrated the outstanding post-washing performance, were maintained the microbial reduction rates of 72.6%, 69.4%, and 69.6% after 5 washing cycles, and 59.0%, 59.2%, and 61.2% after 10 washing cycles. These results confirm the durability and effectiveness of PdNPs-functionalized cationic viscose fabrics, making them promising candidates for application as long-lasting microbicide textiles in healthcare and hygiene-related application.

Microbial protective textiles were recently manufactured via the immobilization of various metallic particles such as silver (Ag), gold (Au) [24, 34, 35, 98–103], hybrid systems like Ag@natural dye and Pd@natural dye [23, 29], metal oxides including TiO₂, ZnO, and Cu₂O [22, 104–110], and metal-organic complexes [86, 111–113]. The microbicide performance of these materials is consistent with the findings presented in this approach, regardless to the used measurement techniques. The mechanism underlying the microbicide action of the Pd-modified viscose fabrics is primarily attributed to the generation of ROS (reactive oxygen species) [114–121]. The immobilized PdNPs are capable of generating ROS such as singlet oxygen and hydroxyl radicals, which can act for inducing microbial cell death. ROS can penetrate microbial cell walls, trigger oxidative stress, and inhibit genetic replication. Additionally, ROS may show dysfunction of mitochondrial, peroxidation of lipid, and structural damaging to the cell wall through necrotic or apoptosis, ultimately resulting to the complete destruction of microbial cells.

By comparing PdNPs to other commonly used NPs (Ag, TiO₂) in terms of cost or environmental impact, PdNPs shows outstanding UV blocking with high transparency and minimal concentration, high durability and non-photo-catalytic action (unlike TiO₂, which can decompose the textile matrix), environmentally safer than AgNPs, which are toxic to aquatic ecosystem, and more suitable for multi-functional textile applications (e.g., wash durability, photo-stability). Moreover, Palladium as a metal is chemically stable and does not readily solubilize or releasing ions under ambient conditions, to show better durability and lower risk of toxic impact from releasing [122].

## Conclusion

PdNPs are increasingly identified for their innovative applicability in the textile industrialization, attributing to their unique physicochemical characters and multifunctional potentiality. These nano-scaled particles exhibit exceptional thermal and chemical stabilization, alongside observable UV-resistance and antimicrobial capabilities, to be suitable for application in textile industries that meet modern demanding for durability and functionality. In this approach, an effective technique was functionalized via the immobilization of PdNPs onto viscose fabrics via in-situ reduction using an IR-assisted approach. Characterization through SEM–EDX, FT-IR, XPS, and XRD was performed for confirmation of the successful deposition of PdNPs on the cellulosic matrix. The treated fabrics showed remarkable ultraviolet protection, as indicated by UPF values of 88.0 for Q-Vs-IR-2 and 50.4 for Vs-IR-2, with durability sustained even after 5 and 10 washing cycles (66.1 and 48.0, respectively, for Q-Vs-IR-2). Additionally, the Q-Pd-viscose samples were shown with excellent antimicrobial action against *Staphylococcus aureus*, *Escherichia coli*, and *Candida albicans*, achieving up to 93.2%, 90.5%, and 94.3% inhibition, respectively. Observably, strong antimicrobial action was retained after 5 and 10 washing cycles, maintaining reduction % of 72.6%, 69.4%, and 69.6%, and 59.0%, 59.2%, and 61.2%, respectively. These data highlight the dual functionalization and durability of PdNPs-treated viscose fabrics, making them as promising candidates for high-performance antimicrobial and UV-protective fabrics.

## Supplementary Information

Additional file 1.

## Data Availability

All relevant data are within the manuscript and available from the corresponding author upon request.
