# Supplementary material for: Sustainable infrared-driven deposition of palladium nanoparticles on viscose for multi-functional textile engineering
Source: BMC Chem. 2025 Dec 10;20(1):9. doi: 10.1186/s13065-025-01690-0 (PMC12801605; doi:10.1186/s13065-025-01690-0)
Supplement: Supplementary file 1 — Additional file 1. [file 13065_2025_1690_MOESM1_ESM.docx]

**Supplementary file**

**Figure S1:** Scanning microimages, EDX analysis and mapping for the functionalized cationized viscose fabrics (Q-Vs-IR-2); **[a]** 5 washings and **[b]** 10 washings.

**
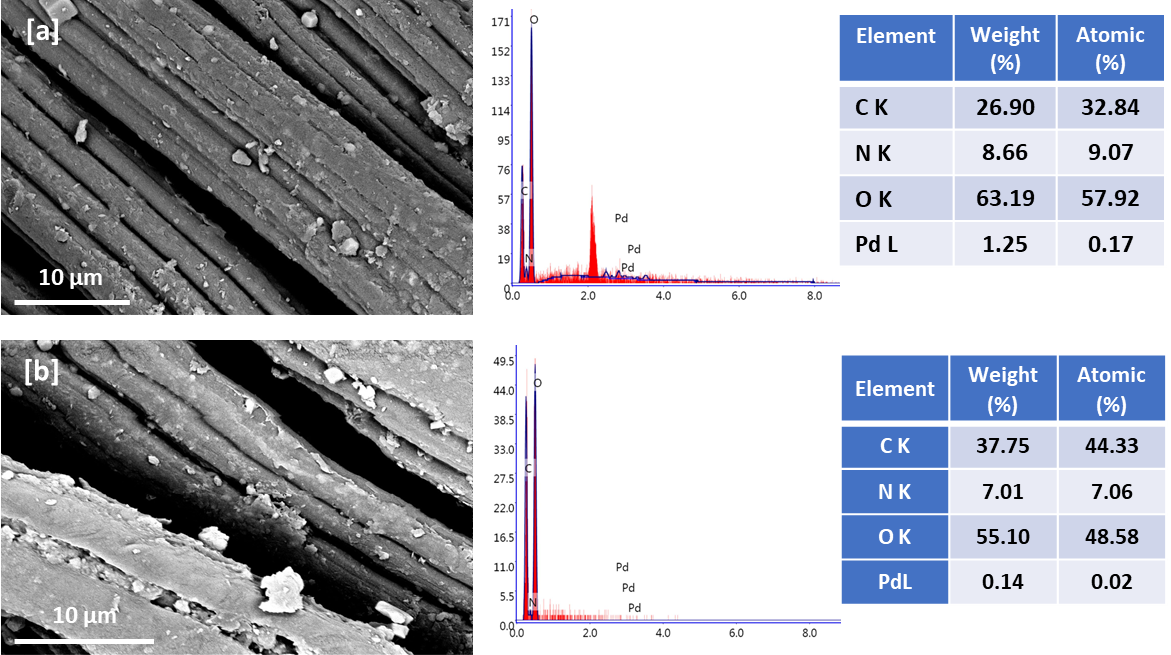
**

**Figure S2:** Color strength for the functionalized viscose fabrics after washings; **[a, b]** 5 washings and **[c, d]** 10 washings.

**
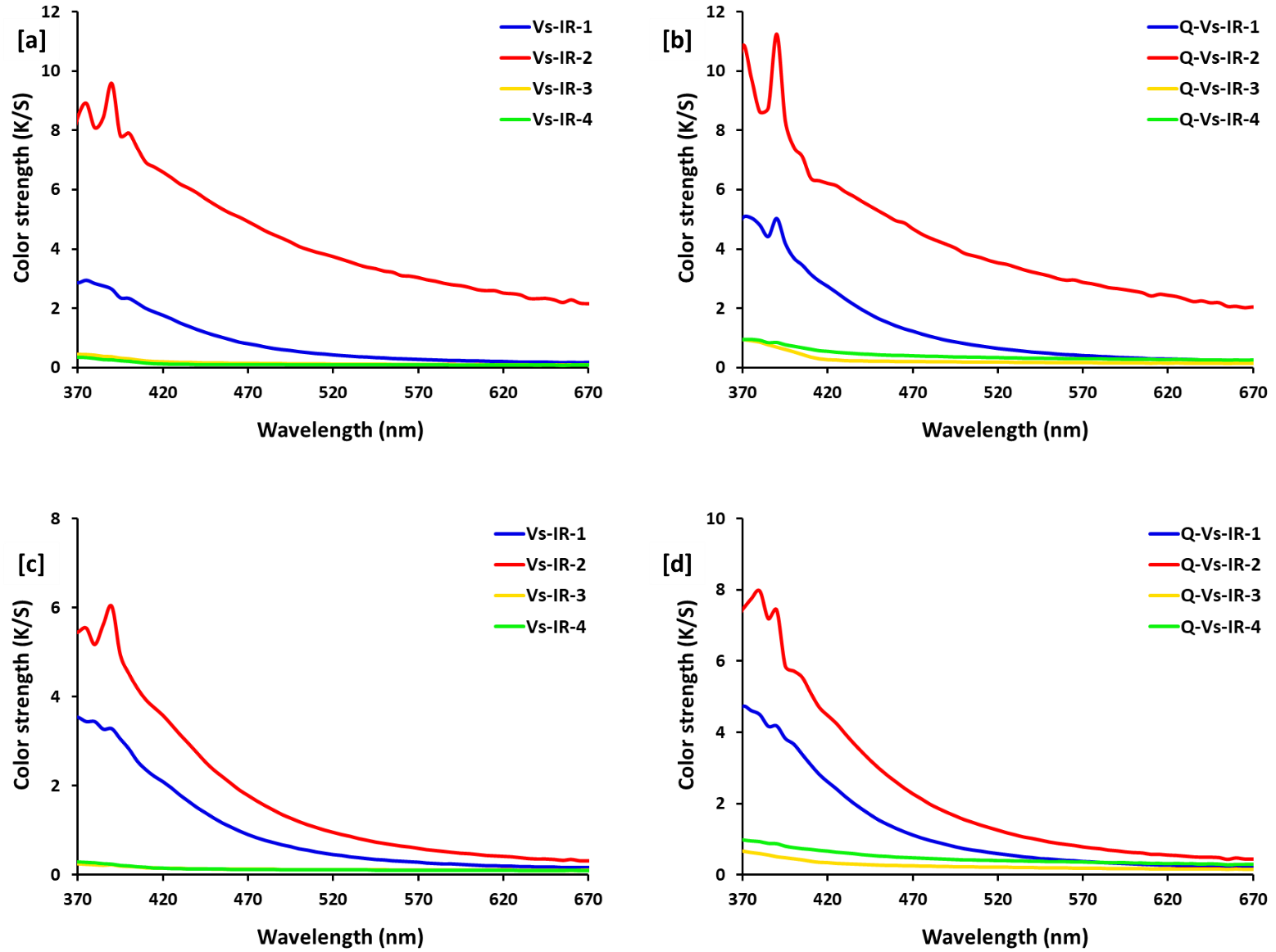
**

**Figure S3:** Absorbance for the functionalized viscose fabrics after washings; **[a, b]** 5 washings and **[c, d]** 10 washings.


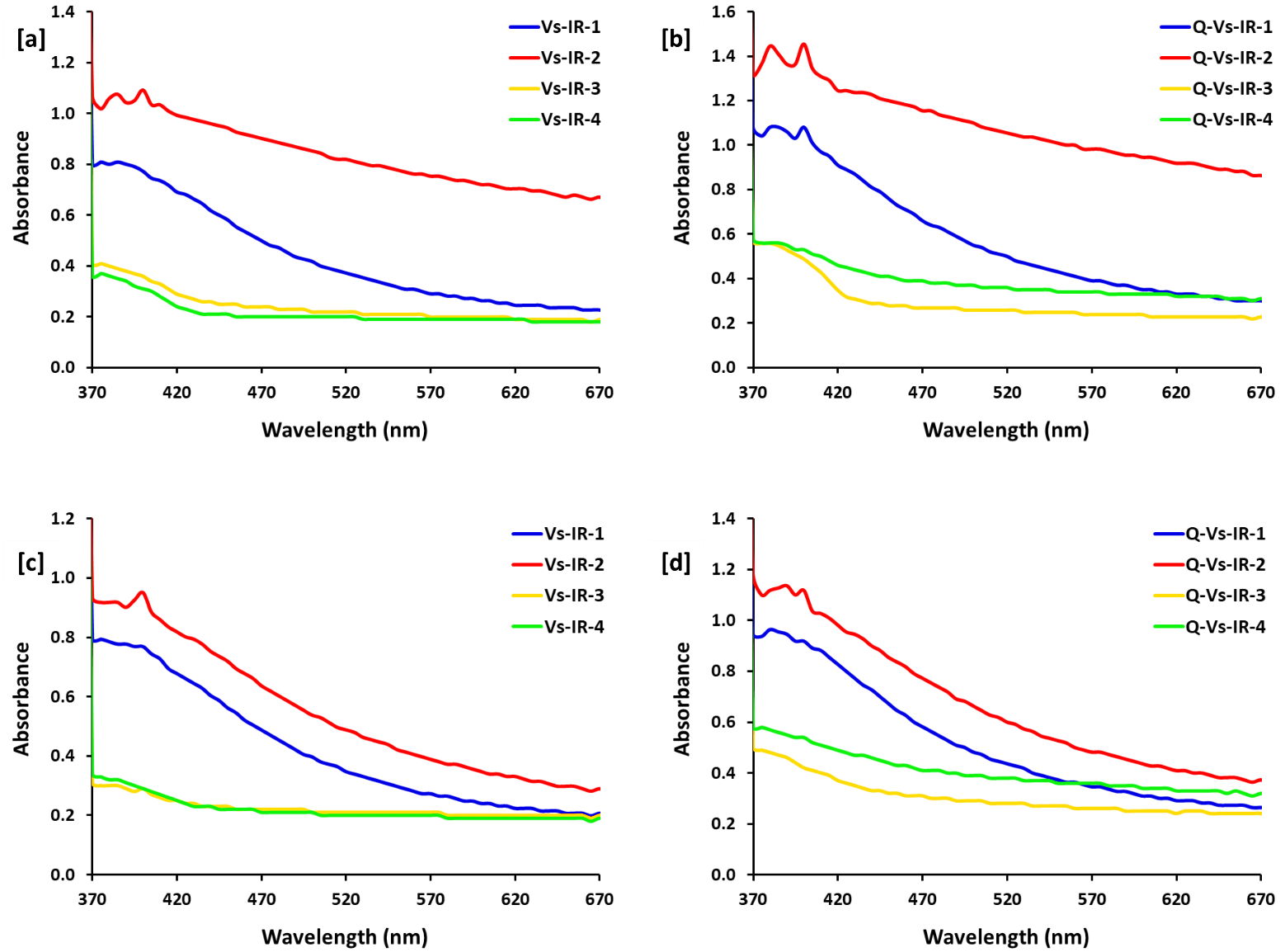


**Figure S4:** Ultraviolet radiation (UVR) transmission through the functionalized viscose fabrics.

**
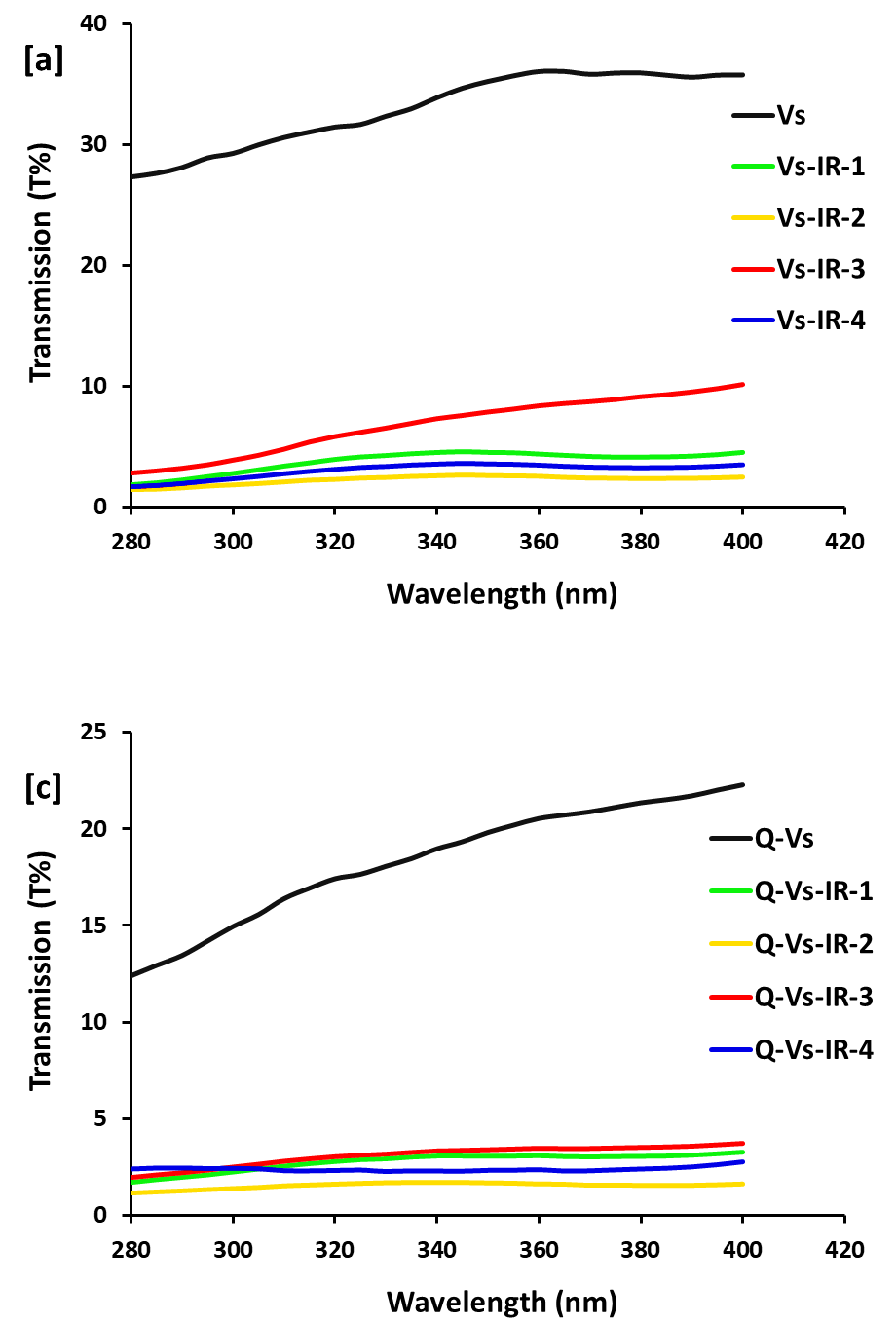
**
